# The Clinical Pathway Initiative: Identifying role relevant competencies in genomic pathways

**DOI:** 10.1002/jgc4.70170

**Published:** 2026-06-04

**Authors:** Lianne Gompertz, Danielle Bogue, Judith Hayward, Melanie Watson, Edward Miller, Kate Tatton‐Brown

**Affiliations:** ^1^ National Genomics Education Programme NHSE Birmingham UK; ^2^ Manchester Centre for Genomic Medicine Manchester UK; ^3^ Affinity Care Bradford UK; ^4^ NHS Genomic Services in the South West Bristol UK; ^5^ St. George's University of London London UK; ^6^ St George's University Hospital NHS Trust London UK

**Keywords:** clinical pathway, competencies, genomic, mainstreaming

## Abstract

Mainstreaming genomic medicine creates the challenge of ensuring confidence and competence in the healthcare workforce. The Clinical Pathway Initiative (CPI), led by the NHS Genomics Education Programme, maps competencies and educational resources to clinical pathways where genomics is used to deliver care. This approach supports integration of genomic medicine through a consistent, workforce‐agnostic, competency‐based framework. Using a familial hypercholesterolemia (FH) clinical pathway, we conducted a process evaluation to assess the usefulness, usability, and acceptability of the CPI in both development and implementation. Quantitative data were collected via rating scales and a modified System Usability Scale (SUS), while qualitative data were generated from interviews and short‐answer survey questions with participants, including a range of professional roles and individuals involved in both development and implementation. SUS scores were 65 for development and 72.5 for implementation (68 or higher is above average). Overall, participants recognized the initiative's value in supporting workforce competence. Interviewees highlighted its simplicity, transferability, and role in promoting consistent, high‐level genomic standards. These findings support ongoing refinement and evaluation of the CPI in real‐world practice.

**What is known about this topic**

Mainstreaming genomic medicine for improved patient care requires genomic education and training at scale and pace.

**What this paper adds to the topic**

The Clinical Pathway Initiative (CPI), led by the Genomics Education Programme within NHS England, aims to identify the competencies required to deliver genomic healthcare via a national and consistent approach. This process evaluation of the first CPI to be developed demonstrates its potential in supporting the wider workforce to navigate and integrate genomic competencies into mainstream practice.

## 1 INTRODUCTION

Over the past 10 years, there has been massive and rapid expansion of genomic practice across the globe (Stark et al., 2019). In England, the NHS Genomic Medicine Service (GMS) is driving the delivery of genomic medicine into routine care (NHS England, 2022a). To facilitate equitable access to genomic testing, NHS England (NHSE) has launched its National Genomic Test Directory (NHS England, 2022b), encouraging a wider range of specialties outside of Clinical Genetics to undertake genomic testing. It is therefore essential that healthcare workers are provided with role‐specific genomic education and training (Martyn et al., 2022; NHS England, 2022a). The Clinical Pathway Initiative (CPI) was developed by NHSE's Genomics Education Programme (GEP) with the aim to facilitate the integration of genomic medicine across healthcare specialties and professions. This manuscript outlines the methodology of CPI development, presents initial findings of a process evaluation, and aims to explore the acceptability, usefulness, and usability of the framework in its ability to outline genomic competencies in mainstream genomic clinical pathways.

## 2 APPROACH/STEPS TAKEN FOR DEVELOPMENT AND IMPLEMENTATION

**FIGURE 1:**
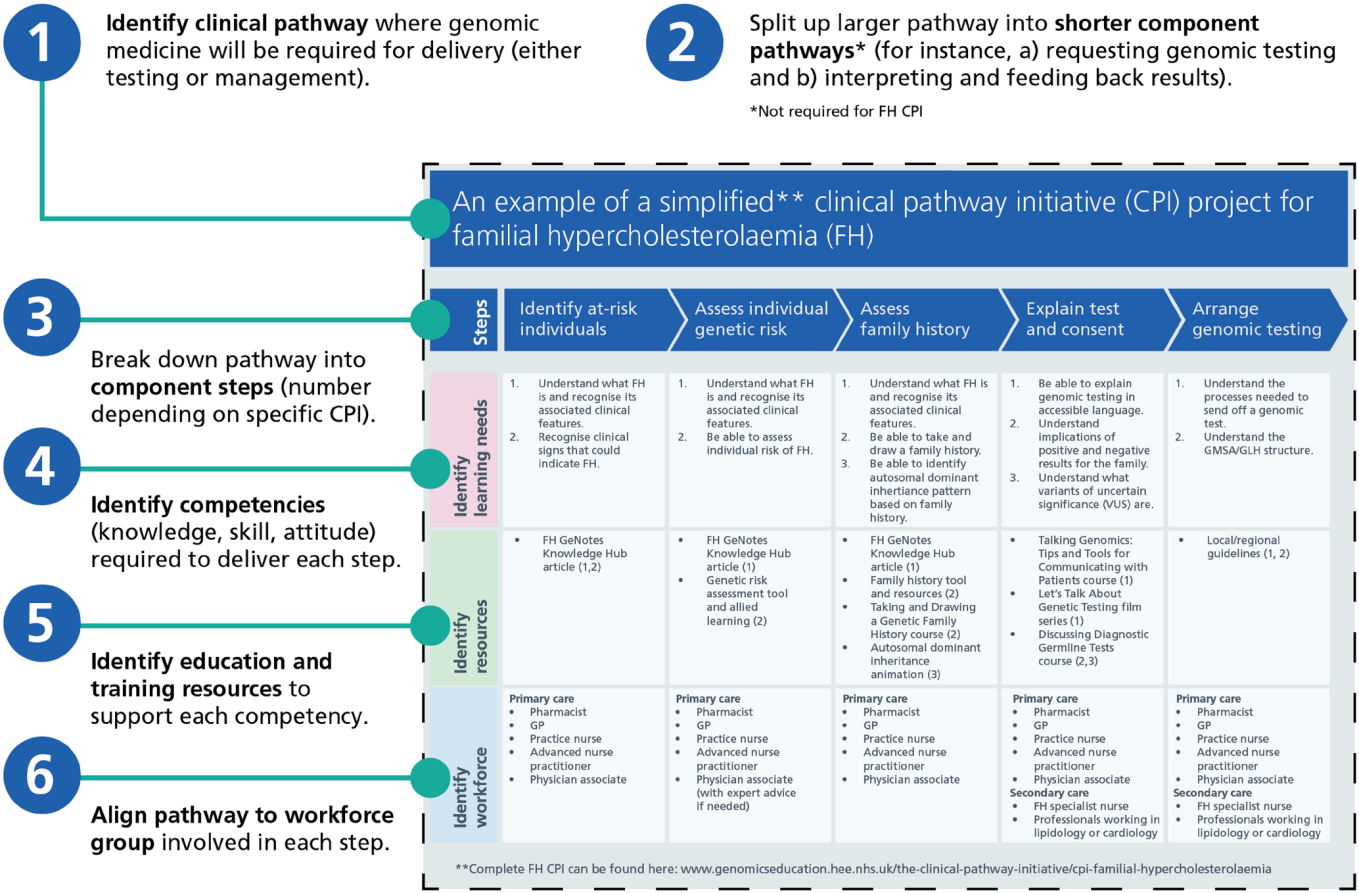
Process of creating a CPI pathway: First, a genomics‐based clinical pathway is identified; the pathway is then sub‐divided into component steps (e.g., requesting a test, return of results, etc) which are, in turn, mapped to the competencies required to deliver each step. A gap analysis is undertaken for educational resources, and, if necessary, education and training resources are developed to meet the competencies. Finally, each step is aligned to the likely workforce group (profession and specialty) delivering the step. After careful review by expert clinical, educational, and editorial members of the GEP, CPIs are published to the website (NHS England, 2022c) to be freely accessed.

A CPI is a high‐level educational resource designed to reflect contextual learning theory (Martyn et al., 2022); that being, learning is optimized when delivered in the context through which it should be applied. Genomic competence aligned to service delivery models has been proposed as one enabler to promote efficient and effective education for the wider workforce (Nisselle et al., 2023). The CPI is designed to allow individuals to personalize genomic education and training that is relevant to their role and professional development. The process of developing a CPI, and an example of a simplified CPI, is shown in Figure 1.

Thirteen CPIs have so far been published (NHS England, 2022c). The first of these was to support investigation of familial hypercholesterolemia (FH) (from specialist lipidology services to primary care) in a routine care setting. FH affects approximately 1 in 220 people and increases predisposition to cardiovascular disease (Sarraju & Knowles, 2019). Given its prevalence and health benefits of early diagnosis (Wiegman et al., 2015), NHSE had identified FH as a priority for the Genomic Medicine Service (NHS England, 2022a). The FH CPI was published in August 2023 (Genomics Education Programme, NHS England, 2022a) and has since acted as an exemplar for CPI development and implementation.

## 3 EVALUATION OF INNOVATION

To guide the evaluation of the CPI, we developed a logic model to map how the educational intervention was expected to achieve desired outcomes (Nisselle et al., 2019). Using this model, we conducted a process evaluation between April and May 2023. Process evaluations are particularly valuable for exploring how change occurs within complex interventions by capturing contextualized information about who delivers the intervention, what is delivered, when, and under what circumstances (Skivington et al., 2021). Grounded in a pragmatic research paradigm, the evaluation combined qualitative and quantitative methods to generate a comprehensive understanding of how the CPI was perceived and used in practice.

Case studies are widely regarded as a suitable methodological approach for process evaluations (Grant et al., 2020). The FH CPI was selected as an initial case due to its clear actionability and established protocols in both primary and tertiary care. This enabled exploration of contributors' experiences in authorship and implementation, offering a real‐world view of how a CPI can be developed, applied, and refined to support broader adoption. The evaluation focused on three key domains—usefulness, acceptability, and usability—which are critical for understanding engagement with this new educational tool and informing its further development.

All FH CPI authors (*n* = 15) were invited to participate. At the time of evaluation, the CPI had not been widely published, and only two end users were known to be using it. Despite this, their input was considered valuable in capturing early user experiences and recognizing the typical lag between innovation and widespread adoption.

To support a comprehensive understanding of the CPI, a convergent mixed‐methods design was employed (Borle & Austin, 2025). This approach enabled the integration of qualitative and quantitative data to assess not only measurable outcomes (e.g., perceived usability), but also the contextual and experiential factors influencing those outcomes.

**FIGURE 2:**
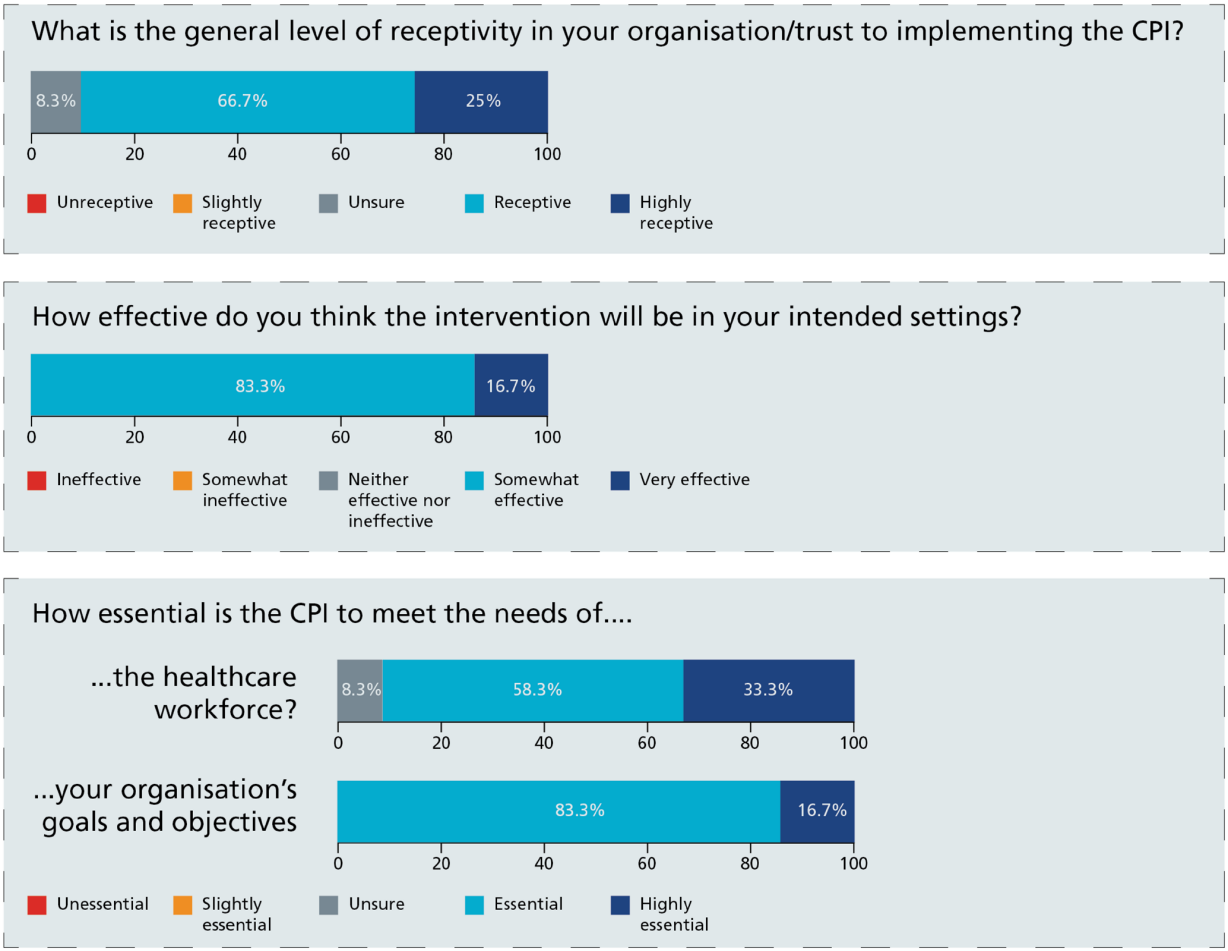
Survey rating scales and responses for authors of the CPI.

Quantitative survey data were obtained based on an adapted 10‐question “System Usability Scale” (SUS) (Brooke, 1996; Appendix S1). The SUS is a validated and reproducible tool, which provides an assessment of overall usability. This encompasses effectiveness (i.e., how CPI authors and end users can successfully write or use a CPI), efficiency (how much effort is expended through this activity), and satisfaction (was the overall experience of writing or using the CPI satisfactory). An SUS score of 68 indicates above average usability. SUS scores were analyzed to determine usability during the CPI writing process and usability of the completed framework for implementation as experienced by end users. Additional scores from questionnaire rating scales (Figure 2) also supplemented quantitative data.

Qualitative data were collected through two approaches. First, short‐answer feedback was requested from FH CPI authors (Appendix S2); end users were not asked to complete this to avoid duplication in the small sample. Second, semi‐structured interviews were conducted with four lead authors and both known end users, selected through purposive sampling based on central roles in the design, authorship, or implementation of the FH CPI. Guided by the concept of information power (Malterud et al., 2015), this targeted sampling approach ensured rich and relevant insights from a small, focused group.

Interview questions were adapted from the Consolidated Framework for Implementation Research (CFIR) Interview Guide Tool (CFIR Research Team, 2022), which offers constructs known to influence implementation. The adapted guides are included in Appendix S3. Interviews explored participants' experiences in writing or using the CPI, confidence in the tool, and other factors relevant to effective implementation.

Using the Framework Method (Gale et al., 2013), two members of the evaluation team conducted initial unrestricted coding, followed by refinement and categorization into overarching themes of usefulness, acceptability, and usability. Efforts were made to preserve contextual nuance by linking each theme to broader aspects of participants' narratives, a common strategy in qualitative health research (Pope et al., 2000).

Quantitative and qualitative data were collected concurrently, with equal priority given to both. Integration occurred during interpretation, where qualitative themes were compared with quantitative patterns to triangulate conclusions.

This evaluation was exempt from Research Ethics Committee review, as ethical approval was not required for service development and evaluation under local and institutional guidelines.

## 4 RESULTS

Of 15 FH CPI authors invited to participate, 12 completed the survey. Authoring survey respondents represented a range of professional roles, including education and training, project leadership within genomic medicine services, healthcare professionals, and national advisory roles. Participants were recruited from multiple implementation settings across the country.

Interview participants reflected this breadth of experience. End users included pharmacists working in different practice contexts, capturing perspectives from applied use of the CPI across distinct implementation environments.

### 4.1 Usefulness: Does the CPI provide an effective educational framework to support the mainstreaming of genomic competencies?

The evaluative results emphasized the initiative's significant value and relevance in supporting genomic learning objectives. In meeting the needs of the mainstream workforce, 11 of 12 authoring survey responders rated the CPI as essential or highly essential to meeting the needs of the workforce in mainstreaming genomic medicine. All respondents rated the CPI as essential or highly essential to meeting the needs of their organization (healthcare service, educational body, or charity; Figure 2). This was enriched by qualitative responses:

Interviewee 1 “…it's essential to have a competency framework for any mainstream genomic test… you're asking (other professionals) to do something, which has been the remit of the clinical genetic specialty for…years.”

The potential of the CPI to support genomic competence within professional roles was also recognized, particularly through its ability to facilitate consistent, evidence‐based standards.

Survey Responder 1 “It provides a consistent approach to teaching and allows learners to appreciate the patient journey.”

Interviewee 4 “One of the key benefits of CPIs is to provide a standardization across different multidisciplinary workforce groups so that there's almost a standard of high‐quality care no matter what your professional occupational role.”

Standardization of competencies and clinical governance was felt to be an important use of the CPI in role specification, job planning, and service development.

Interviewee 3 “I feel there's quite a lot of variation in practice.”

Interviewee 5 “The CPI definitely provides assurance for service commissioners to know exactly what's being provided (and) know that there's consistency in what's being provided.”

The CPI was felt to raise awareness of more complex issues surrounding genomic consent and counseling. This may be a new skill for end users, which may require supporting resources.

Interviewee 5 “...genetic counseling and gaining consent [is] a very new skill.”

Interviewee 2 “...the framework really helps with understanding those complex issues (ethical issues and… families not getting along) … issues that you might never have had to deal with before.”

The correct targeting of training was also considered to allow for role optimization while being financially efficient.

Interviewee 1 “Pharmacists already had a considerable role in managing lipids …it seemed a natural extension of their role.”

Interviewee 6 “...(pharmacists) are expensive in comparison to nurses… it's (about) making sure …the use of your whole workforce is done appropriately.”

### 4.2 Acceptability: To what extent is the CPI a suitable educational framework to support the attainment of genomic competencies in the mainstream?

Engagement and willingness to adopt a new educational initiative is influenced by both individual and environmental factors, which are critical to the success of an intervention. In this evaluation, 11 of 12 survey respondents and both end users reported being *receptive or highly receptive* to the CPI (Figure 2). Consistent with previous research (CFIR Research Team, 2022), some interview participants identified a lack of understanding and familiarity with genomics as ongoing barriers to the adoption of genomic practice. However, the CPI was viewed as a promising resource to help overcome these challenges.

Interviewee 4 “In my opinion, I don't think my local colleagues are aware of genomic medicine.”

Interviewee 1 “I think to be able to change your attitude towards genomics and genetics, [the CPI] would be a good motivator.”

Survey responders also noted that the CPI could “empower professionals to feel more competent” and reduce reluctance to engage with genomics.

Participants reflected on the familiarity, and thus transferability, of the CPI as an educational framework to apply in their setting.

Interviewee 5 “...(there are) competency frameworks, which are similar…however, nothing exists at the moment for genomics.”

Interviewee 1 “...and certainly the allied healthcare professionals… it very much fits with their working practices that they would (need).”

Survey response 4 “...(the) CPI can be used to underpin the development and delivery of education initiatives (and) to allow for assessment purposes if required. The CPI could also be applied to direct or influence professional and service development.”

### 4.3 Usability: Is the CPI an effective, efficient, and satisfactory method to support the mainstreaming of genomic competencies?

Ease of usability underpins engagement, efficiency, and satisfaction and is imperative to sustained adoption of a new practice.

Ten of the twelve authoring survey respondents of the FH CPI and both end users completed the System Usability Scale (SUS) component of the survey. The median score for the authoring group was 65 (range: 42.5–90) and 72.5 (range: 62.5–82.5) for implementation. Interviewees summarized the ease of usability:

Interviewee 2 “...[the CPI is] as straightforward and simplistic as possible.”

Adaptations to the original CPI template were also easily integrated to enhance the usability of the FH CPI for end users.

Interviewee 3 “We did also identify that it would be useful to have a further step, which was around supporting data and digital solutions for each step as well.”

Considerations to improve the format of the CPI and overcome potential barriers to successful implementation, and activities consequently undertaken to address them, are represented in Table 1.

**TABLE 1:** Key insights derived from thematic analysis of participant interviews and open‐text survey responses, alongside corresponding activities undertaken by NHSE Genomics Education Programme in response to these findings, to support and improve the implementation of the CPI.

	Summary of insight	Representative quotations	NHSE genomics education activities
Lack of awareness	Awareness of the CPI and NHSE Genomics Education Programme needs to be increased	Participant 6: “it feels for me that genomics is still a very specialist service and perhaps only specialists would look for it or know where to look for it.” Survey Responder 4: “Mainstream users may be unaware of the Genomics Education Programme… (we) need to that the ensure CPI is appropriately sign posted on partner websites.”	The CPI and other educational resources designed and developed by NHSE Genomics Education Programme have been presented at national conferences and advertised through specialty‐specific Royal Colleges within the UK. Sharing of success stories in developing and using CPI projects will be published via the CPI webpage in due course.
Practicality with visualization	Presenting the CPI as an excel spreadsheet is suboptimal to reviewers and end users.	Survey Responder 5: “I think the excel document for the CPI can be difficult to use and visualize” Participant 5: “Simplifying the CPI content format and design for practical use will simplify it and make it more attractive for the user's eye to process. This has influenced some of the (SUS) scores”	Completed CPI projects visualized on the webpage with vertical drop‐down menus for aligned educational resources in each step and optional downloadable spreadsheet.
Overarching comments	Numerous competencies can be applicable to multiple CPI steps or pathways	Participant 2: “we recognized that there were overarching competencies that would apply to the whole of the CPI and started to think about how they could be represented within the CPI.” Survey Responder 6: “A lot of the genomics side of the CPI is generic and transferable”	A new CPI template has been created with pre‐filled competencies that are relevant to most CPI projects. A column added to new template entitled “Fundamental principles relevant to all steps” to avoid duplication within the template (Pichini et al., 2024).
Adaptations of the CPI	The CPI can be adapted to need.	Participant 2: “We did also identify that it would be useful to have a further step, which was around supporting data and digital solutions for each step as well.”	In the case of the FH CPI, a “digital solutions” column was added to compliment the outlined competencies by using standard applications/programmes used by general practice within England. Other considerations include role/grade separation and inclusion of a self‐declaration of competencies.
Authoring group support	Further clarity and direction may be helpful for future CPIs	Survey responder 2: “(support in writing) may be required from the GEP team.” Survey Responder 1: “A ‘quick guide’ including how to word learning objectives/educational needs would be helpful.”	The Genomics Education Programme have generated an improved, easy to navigate CPI webpage with provision of authoring guides (Pichini et al., 2024). Online workshops and drop‐in sessions are also held on a regular basis for authors.

## 5 DISCUSSION

This evaluation has demonstrated that the CPI is perceived as a useful, acceptable, and usable tool to identify and support the attainment of genomic competencies in the wider workforce. The evaluation highlighted elements of the method that could improve usability for those authoring a CPI.

Results from our evaluation reference the importance of standardization of genomic competencies for role specification, job planning, and service development, supporting the NHSE “Accelerating genomic medicine in the NHS” strategy (NHS England, 2022a). Competency frameworks have previously been used to guide clinicians in the communication of genetic results (A‐Hallquist et al., 2021; Pichini et al., 2024). The CPI builds upon this to include the pre‐test setting and refinement to set patient pathways. Results from our evaluation align with published literature suggesting that a competency framework is an acceptable and familiar format for healthcare professionals (Harding et al., 2024; NHS England, 2023; Tonkin et al., 2018).

Potential barriers to successful CPI implementation, such as workforce capacity and time (leading to workforce resistance to incorporating genomics into practice), funding, and lack of awareness of genomic role relevance and/or education initiatives, were reflected upon by participants and are well represented in current literature (McClaren et al., 2020; Nisselle et al., 2023; Raspa et al., 2021; Simpson et al., 2019; Zebrowski et al., 2019). A reluctance to accept genomics as an active role requirement for wider specialists has been previously reported (White et al., 2020). To overcome this, the CPI is designed to contextualize genomic relevance by keeping the patient pathway and thus the role of the healthcare provider, at its focus. An appreciation of how a service or intervention (genomic medicine) can be used to transform healthcare, as well as its relevance to the role of an individual healthcare professional, is recognized to motivate behavioral change (McClaren et al., 2020; Simpson et al., 2019).

It is acknowledged that increased complexity may be involved in other genomic pathways. The CPI serves to support accessibility to genomic testing and builds upon awareness of these complex issues. Implications of genomic testing relating to types of result (including variants of unknown significance) and ethical considerations have been highlighted in subsequent resources created for pathway development (Genomics Education Programme, NHS England, 2022b, 2023) following this evaluation to ensure appropriate scope and depth. Clinical genetic services have historically addressed many workforce development challenges that the CPI now seeks to systematize, and CPI working groups are encouraged to include clinical genetics professionals when developing a pathway. CPIs also highlight the requirement to work within one's scope of practice, with appropriate liaison and referral to Clinical Genetics departments in circumstances where this may exceed the skill of the mainstream professional.

While findings are promising, they are based on a small, self‐selected sample and should be interpreted as preliminary. The focused evaluation scope, CFIR‐informed interview questions, and consistency across responses supported the adequacy of the sample for meaningful analysis. Participants generally viewed the CPI project positively, though findings were reported objectively and potential biases acknowledged. Several authors of this manuscript are members of the GEP team responsible for delivering the CPI, including one involved in authoring the FH CPI. These insider roles provided valuable context but may have introduced bias; to mitigate this, we employed structured data collection, collaborative coding, and triangulation across qualitative and quantitative sources. One participant's SUS responses differed from the majority, potentially reflecting limited involvement in the writing process. Nevertheless, convergence of data across methods supports the utility of the innovation and highlights areas for refinement, particularly from an authorship perspective.

Results from this process evaluation have already helped to improve the CPI method and informed a strategy for a longer‐term real‐world evaluation. The NHSE Genomics Education Programme has developed an “evaluation toolkit” of prepared and validated tools used in genomic medicine and education for authors and end users of individual CPI projects, pre‐ and post‐CPI implementation, to better understand the impact of the initiative on workforce education, training, and ultimately patient care. Imperative to ensuring best practice in genomics education and evaluation, the GEP team recommend that reporting of any educational intervention should reflect new standardization as outlined by RISE2 Genomics (Nisselle et al., 2021).

To conclude, the CPI has potential to support the role of genetic counselors and clinicians by embedding their expertise within clearly defined, multidisciplinary care pathways. By standardizing best practices, the initiative helps to clarify referral processes and enhance the visibility and impact of the genetic counselor's role. The Clinical Pathway Initiative could also offer an opportunity to support the educational role of genetic counselors in upskilling the wider workforce for genomic mainstreaming by providing a framework through which they can share their specialist knowledge with other healthcare professionals.

## AUTHOR CONTRIBUTIONS

**Lianne Gompertz:** Conceptualization; data curation; formal analysis; investigation; methodology; validation; writing – original draft. **Danielle Bogue:** Data curation; formal analysis; validation; writing – review and editing. **Judith Hayward:** Conceptualization; methodology; writing – review and editing. **Melanie Watson:** Methodology; writing – review and editing. **Edward Miller:** Conceptualization; methodology; supervision; writing – review and editing. **Kate Tatton‐Brown:** Conceptualization; supervision; writing – review and editing.

## CONFLICT OF INTEREST STATEMENT

The authors Dr. Lianne Gompertz, Dr. Danielle Bogue, Dr. Judith Hayward, Melanie Watson, and Edward Miller had roles within the Genomics Education Programme at the time of this evaluation. Author Dr. Kate Tatton‐Brown is the Clinical Director and head of the National Genomics Education Programme.

## ETHICS STATEMENT

Human Studies and Informed Consent: All participants in this evaluation consent to publication.

Animal Studies: Statement is not applicable for this submission.

## Supporting information


Appendix S1.

## Data Availability

Data are available upon request.
