# Supplementary material for: The Clinical Pathway Initiative: Identifying role relevant competencies in genomic pathways
Source: J Genet Couns. 2026 Jun 4;35(3):e70170. doi: 10.1002/jgc4.70170 (PMC13238389; doi:10.1002/jgc4.70170)
Supplement: Supplementary file 1 — Appendix S1. [file JGC4-35-0-s001.zip › Appendix 1 new.docx]

Appendix 1: Adapted System Usability Scale for authors involved in writing the CPI pathway

On a Scale of 1-5, (1 being strongly disagree and 5 being strongly agree), please could you rank the following statements in relation to the Clinical Pathway Initiative.

1. I think that I could write CPIs frequently.
2. I found writing the CPI unnecessarily complex.
3. I thought that the CPI writing process was easy.
4. I think that I would have liked more support from the GEP team to write this CPI.
5. I found the various processes to write this CPI well integrated/streamlined.
6. I thought that that was too much inconsistency in writing this CPI.
7. I imagine that most people could write a CPI very quickly.
8. I found writing the CPI very cumbersome.
9. I felt very confident in writing the CPI.
10. I needed to learn a lot of things before I could write a CPI.

Adapted System Usability Scale for end users of the CPI pathway

On a Scale of 1-5, (1 being strongly disagree and 5 being strongly agree), please could you rank the following statements in relation to the Clinical Pathway Initiative.

1. I think that I would like to use CPIs frequently.
2. I found the CPI unnecessarily complex.
3. I thought that the CPI was easy to use.
4. I think that I would need the support of the CPI author to use this CPI.
5. I found the various competencies and steps in this system were well integrated/streamlined.
6. I thought that there was too much inconsistency in content of this CPI.
7. I would imagine most people would learn from this CPI very quickly.
8. I found the CPI very cumbersome to use.
9. I felt very confident using the CPI.
10. I needed to learn a lot of things before I could get going with this CPI.

To score the SUS:

- For odd items, subtract 1 from the user response
- For even numbered items, subtract the user responses from 5
- Add the concerted responses for each user and multiply by 2.5.
