# Supplementary material for: The Clinical Pathway Initiative: Identifying role relevant competencies in genomic pathways
Source: J Genet Couns. 2026 Jun 4;35(3):e70170. doi: 10.1002/jgc4.70170 (PMC13238389; doi:10.1002/jgc4.70170)
Supplement: Supplementary file 1 — Appendix S1. [file JGC4-35-0-s001.zip › Appendix 2 new.docx]

Appendix 2 Qualitative Survey Questions for authors of the CPI

Would you like to add anything extra about your motivation for experience of developing this CPI?

How do you anticipate this CPI being used?

Do you anticipate any barriers to implementation of the CPI? Please explain your answer.

Are you aware of any means that have been used to publicise the CPI within your organisation/trust?

Please add any additional comments to support the answers to rating scale questions.
