# Supplementary material for: The Clinical Pathway Initiative: Identifying role relevant competencies in genomic pathways
Source: J Genet Couns. 2026 Jun 4;35(3):e70170. doi: 10.1002/jgc4.70170 (PMC13238389; doi:10.1002/jgc4.70170)
Supplement: Supplementary file 1 — Appendix S1. [file JGC4-35-0-s001.zip › Appendix 3 new.docx]

Appendix 3: interview guides adapted from the CFIR framework^(15)^

Intervention Characteristics:

Why is the intervention being implemented in your setting/Why did you choose this CPI?

**Evidence, Strength and Quality:** What do influential stakeholders think of the intervention

**Relative Advantage:** How does the intervention compare to other similar existing programs in your setting?

**Adaptability:** What kinds of changes or alterations do you think you will need to make to the intervention so it will work effectively in your setting? Are their components which should not be altered?

**Perceived difficulty of implementation, reflected by duration, scope, radicalness, disruptiveness, centrality, and intricacy and number of steps required to implement:** How complicated is the intervention? / Do you think that the CPI framework is complicated to author/use

Outer Setting

**Workforce Needs and Resources:**

To what extent are staff aware of the education and training of the workforce involved in this CPI?

How well do you think the intervention will meet the needs of the workforce identified in your CPI?

**The degree to which an organization is networked with other external organizations.**

To what extent did you network with colleagues or people in similar professions/positions outside your setting when writing this CPI? What kind of information was exchanged?

**External policies and incentives:** What kind of local, national performance measures, policies, regulations, or guidelines influenced the decision to write this CPI?

Inner Setting

**Networks and communications:** Can you describe your working relationship with influential stakeholders?

Were meetings, such as working group meetings, held regularly?

**Culture:**

How would you describe the culture of your working group / the NHS (beliefs, values, assumptions), and how will this affect the implementation of the CPI?  To what extent do you think new ideas will be embraced and used to make improvements.

**Implementation climate:**

What is the general level of receptivity in your working group to implementing the CPI? What is the general level of receptivity in your target service users to implementing the CPI?

Do you feel that there is there a strong need for this intervention?

How essential is this CPI to meet the needs of the workforce and goals of the NHS/Genome Action Plan?

**Compatibility:** How well does the CPI fit with existing work processes and practices in clinical practice? How well does the CPI fit with your values and norms and the values and norms within the NHS/Stakeholder group?

I**ncentives/Rewards** What kinds of incentives are there to help ensure that the implementation of the intervention is successful?

**Goals and feedback:**

Have you/your working group/your organisation set goals related to the implementation of the intervention?

**Available resources:** Do you expect that there are sufficient resources to implement and administer the CPI?

**Characteristics of individuals:**

Do you think the intervention will be effective? (attitude and value placed on the intervention)

**Self-efficacy:** How confident are you that the CPI can be successfully implemented?  How confident do you think your colleagues feel about implementing the intervention?

**Process:**

**Planning-** What did you do to get a plan in place to design/use the CPI? Can you describe the plan for designing/using the CPI?

**Engaging:** What are key stakeholders saying about CPIs? How did you become involved in designing/contributing to the CPI? Will someone (or a team) be helping end users to implement the CPI? What steps have been taken to encourage individuals to use the CPI? How is the CPI being publicised?

**Executing:** How do you anticipate that the CPI will be used?
