# Supplementary material for: The Clinical Pathway Initiative: Identifying role relevant competencies in genomic pathways
Source: J Genet Couns. 2026 Jun 4;35(3):e70170. doi: 10.1002/jgc4.70170 (PMC13238389; doi:10.1002/jgc4.70170)
Supplement: Supplementary file 1 — Appendix S1. [file JGC4-35-0-s001.zip › Appendix.docx]

Appendix 1: Adapted System Usability Scale for authors involved in writing the CPI pathway

On a Scale of 1-5, (1 being strongly disagree and 5 being strongly agree), please could you rank the following statements in relation to the Clinical Pathway Initiative.

1. I think that I could write CPIs frequently.
2. I found writing the CPI unnecessarily complex.
3. I thought that the CPI writing process was easy.
4. I think that I would have liked more support from the GEP team to write this CPI.
5. I found the various processes to write this CPI well integrated/streamlined.
6. I thought that that was too much inconsistency in writing this CPI.
7. I imagine that most people could write a CPI very quickly.
8. I found writing the CPI very cumbersome.
9. I felt very confident in writing the CPI.
10. I needed to learn a lot of things before I could write a CPI.

Adapted System Usability Scale for end users of the CPI pathway

On a Scale of 1-5, (1 being strongly disagree and 5 being strongly agree), please could you rank the following statements in relation to the Clinical Pathway Initiative.

1. I think that I would like to use CPIs frequently.
2. I found the CPI unnecessarily complex.
3. I thought that the CPI was easy to use.
4. I think that I would need the support of the CPI author to use this CPI.
5. I found the various competencies and steps in this system were well integrated/streamlined.
6. I thought that there was too much inconsistency in content of this CPI.
7. I would imagine most people would learn from this CPI very quickly.
8. I found the CPI very cumbersome to use.
9. I felt very confident using the CPI.
10. I needed to learn a lot of things before I could get going with this CPI.

To score the SUS:

- For odd items, subtract 1 from the user response
- For even numbered items, subtract the user responses from 5
- Add the concerted responses for each user and multiply by 2.5.

Appendix 2 Qualitative Survey Questions for authors of the CPI

Would you like to add anything extra about your motivation for experience of developing this CPI?

How do you anticipate this CPI being used?

Do you anticipate any barriers to implementation of the CPI? Please explain your answer.

Are you aware of any means that have been used to publicise the CPI within your organisation/trust?

Please add any additional comments to support the answers to rating scale questions.

Appendix 3: interview guides adapted from the CFIR framework^(15)^

Intervention Characteristics:

Why is the intervention being implemented in your setting/Why did you choose this CPI?

**Evidence, Strength and Quality:** What do influential stakeholders think of the intervention

**Relative Advantage:** How does the intervention compare to other similar existing programs in your setting?

**Adaptability:** What kinds of changes or alterations do you think you will need to make to the intervention so it will work effectively in your setting? Are their components which should not be altered?

**Perceived difficulty of implementation, reflected by duration, scope, radicalness, disruptiveness, centrality, and intricacy and number of steps required to implement:** How complicated is the intervention? / Do you think that the CPI framework is complicated to author/use

Outer Setting

**Workforce Needs and Resources:**

To what extent are staff aware of the education and training of the workforce involved in this CPI?

How well do you think the intervention will meet the needs of the workforce identified in your CPI?

**The degree to which an organization is networked with other external organizations.**

To what extent did you network with colleagues or people in similar professions/positions outside your setting when writing this CPI? What kind of information was exchanged?

**External policies and incentives:** What kind of local, national performance measures, policies, regulations, or guidelines influenced the decision to write this CPI?

Inner Setting

**Networks and communications:** Can you describe your working relationship with influential stakeholders?

Were meetings, such as working group meetings, held regularly?

**Culture:**

How would you describe the culture of your working group / the NHS (beliefs, values, assumptions), and how will this affect the implementation of the CPI?  To what extent do you think new ideas will be embraced and used to make improvements.

**Implementation climate:**

What is the general level of receptivity in your working group to implementing the CPI? What is the general level of receptivity in your target service users to implementing the CPI?

Do you feel that there is there a strong need for this intervention?

How essential is this CPI to meet the needs of the workforce and goals of the NHS/Genome Action Plan?

**Compatibility:** How well does the CPI fit with existing work processes and practices in clinical practice? How well does the CPI fit with your values and norms and the values and norms within the NHS/Stakeholder group?

I**ncentives/Rewards** What kinds of incentives are there to help ensure that the implementation of the intervention is successful?

**Goals and feedback:**

Have you/your working group/your organisation set goals related to the implementation of the intervention?

**Available resources:** Do you expect that there are sufficient resources to implement and administer the CPI?

**Characteristics of individuals:**

Do you think the intervention will be effective? (attitude and value placed on the intervention)

**Self-efficacy:** How confident are you that the CPI can be successfully implemented?  How confident do you think your colleagues feel about implementing the intervention?

**Process:**

**Planning-** What did you do to get a plan in place to design/use the CPI? Can you describe the plan for designing/using the CPI?

**Engaging:** What are key stakeholders saying about CPIs? How did you become involved in designing/contributing to the CPI? Will someone (or a team) be helping end users to implement the CPI? What steps have been taken to encourage individuals to use the CPI? How is the CPI being publicised?

**Executing:** How do you anticipate that the CPI will be used?
